# Aging and health among migrants in a European perspective

**DOI:** 10.1186/s40985-016-0036-1

**Published:** 2016-10-26

**Authors:** Maria Kristiansen, Oliver Razum, Hürrem Tezcan-Güntekin, Allan Krasnik

**Affiliations:** 1 Center for Healthy Aging (CEHA), Department of Public Health, University of Copenhagen, Copenhagen, Denmark; 2 Department of Epidemiology and International Public Health, School of Public Health, Bielefeld University, Bielefeld, Germany; 3 Danish Research Center for Migration, Ethnicity and Health (MESU), Department of Public Health, University of Copenhagen, Copenhagen, Denmark

**Keywords:** Aging, Health, Migration, Healthcare delivery, Europe

## Abstract

Population aging and the associated changes in demographic structures and healthcare needs is a key challenge across Europe. Healthy aging strategies focus on ensuring the ability to maintain health, quality of life and independent living at old age. Concurrent to the process of population aging, the demographics of Europe are affected by increased migration resulting in substantial ethnic diversity. In this paper, we narratively review the health profile of the growing proportion of aging migrants in Europe, outline key factors shaping health among this diverse group and consider ways of addressing their healthcare needs.

Although factors shaping aging processes are largely similar across populations, migrant-specific risk factors exist. These include exposure to health risks before and during migration; a more disadvantaged socioeconomic position; language barriers and low health literacy; cultural factors influencing health-seeking behaviours; and psychosocial vulnerability and discrimination affecting health and quality of life. Overall, migrants experience the same morbidity and mortality causes as the native populations, but with different relative importance, severity and age of onset and with substantial differences within and between migrant groups. Little is known regarding health behaviours among aging migrants, although differences in cancer screening behaviours have been identified. Indications of widening health differentials between migrants and native populations with age and informal barriers to quality healthcare for aging migrants are causes of concern.

In conclusion, there is a need for attention to migration alongside other determinants of healthy aging. The diversity in individual characteristics, life course processes and contextual factors shaping aging processes among migrants point to the need for a sensitive and comprehensive approach to policies, practices and research within the field of healthy aging. This is important to accommodate for the needs of the growing number of aging migrants in Europe and counter inequities in health and well-being at old age.

## Background

### Migration within the context of population aging in Europe

Population aging caused by consistently low birth rates and increased life expectancy is stated by the European Commission as one of the greatest economic and social challenges for European societies in the 21st century [1]. With more than 20 % of Europeans expected to be 65 years or over in 2025, and a particularly sharp increase in the proportion of people above the age of 80 years, the demographic structures of European countries are undergoing fundamental changes with implication for all sectors of society, not least the health and social care sectors [2]. The importance of addressing population aging in Europe is underscored by changes in age dependency ratios, with a declining share of the population of working age and rising proportions in need of long-term and often complex health and social care in all European countries [2]. Correspondingly, active and healthy aging has been proposed as a keystone for a sustainable Europe in this context of rapid demographic changes [1, 3]. Through a range of intersectoral strategies focusing on individual and structural factors affecting the ability to maintain health and uphold active and independent living into old age, policies outlined both at the global, regional and country levels seek to ensure sustainable societies in the context of population aging [1, 4, 5].

Healthy populations are an important prerequisite for societal progress and prosperity; however, whereas active and healthy aging has been addressed within the context of overall societal sustainability, less focus has been given to diversity in aging processes and experiences both globally and within European populations [5, 6]. The ability to uphold an active and fulfilling life at old age is shaped by a complex range of individual and contextual factors at play across the lifespan of the individual. Variations in indicators of healthy aging such as functional decline, disease burden and quality of life among aging populations are determined by not only well-recognized factors such as gender, sex and socioeconomic position but also by migration and the age at which migration takes place [5–8]. Concurrent to the process of population aging, Europe has experienced increased immigration and a corresponding rise in ethnic diversity, a process that has been more pronounced in the north-western and some southern countries than in the eastern European countries [9, 10]. For historical, political and geographical reasons, migrant populations differ between European countries [9]. Whereas, e.g. the UK, France and the Netherlands have longer histories of receiving migrants and thus have larger proportions of aging people with different ethnic backgrounds, some north-western European countries, such as Denmark, Sweden and Germany, are only now seeing the aging of the so-called first generation of labour migrants, primarily from Turkey, Pakistan (particularly in Denmark and Sweden) as well as Southern Europe and the Balkans (particularly in Germany). In some countries in southern Europe, such as Spain, Italy and Greece, work migration is limited and recent, as these countries have traditionally been emigration countries. The influx of predominantly younger refugees, in particular to Greece and Italy, and subsequently to northern Europe, has been substantial in recent years [9].

The diversity in characteristics of aging migrants in Europe should be clearly recognized in order to grasp the complexity of migration and aging [5]. For instance, in addition to the groups listed above, aging migrants in Germany include resettlers who are ethnic Germans who return-migrated from the former Soviet Union and often know the German language. The so-called sunset migrants comprise of aging north-western Europeans settling in Southern Europe after retirement, often with little integration into the local society [11]. Aging migrants across Europe therefore represent diverse characteristics ranging from the very affluent and privileged groups of ‘sunset migrants’ expressing positive perspectives on active aging to more deprived groups of, e.g. aging refugees with partly or largely unmet needs in terms of health and welfare [12]. Disregarding the great diversity among aging migrants in Europe may result in the construction of an undue homogenous social category of ‘aging migrants’, thus contributing to other discourses on migrants [13].

The ability to reach the goal of healthy aging is shaped by both previous and current individual, social and contextual factors as well as country-level policies and services in the fields of migration, social welfare and health [6, 13, 14]. Overall, migrants, encompassing both labour migrants, asylum seekers and refugees, are younger than indigenous populations in Europe, and although few data have so far been released with respect to the proportion of aging migrants in Europe, an increase can be expected as these groups age and with little indication of remigration [10, 15]. With substantial, persistent differences in health outcomes and their underlying social determinants across different socioeconomic and migrant/ethnic groups [16–19], there is now a need for more focus on how these intersecting characteristics shape healthy aging and ways of addressing this within healthcare services. Insight into the health of aging migrants in Europe is integral to such efforts. To contribute towards this goal, we narratively review the health profile of aging migrants in Europe, outline some of the factors shaping their health and consider ways of addressing their healthcare needs.

## Identifying key literature on aging and migration

Studies for this narrative review were included based on a literature search in the following databases: PsycINFO, MEDLINE and Google Scholar in May 2016 using variations and combinations of the search terms: migrants, migration, aging, aged, health behaviour, morbidity, mortality, health status, mental health, quality of life, health promotion, disease prevention, health services, quality of care, palliative care and remigration. There were no restrictions in terms of year of publication. Only published, peer-reviewed literature in English or German was included in this review. We retrieved a total of 663 publications after de-duplication. The first author screened publications first by title and then by abstract according to the following inclusion criteria:

- Population characteristics: we were interested in studies in migrant populations above the age of 50 years living in Europe
- Outcome: health behaviour, morbidity, mortality, quality of life, healthcare access and utilization

References within relevant articles were screened for additional studies. Full-text versions of relevant abstracts (*n* = 33) and book chapters were obtained for inclusion and summarized and synthesized qualitatively. Key European policies pertaining to healthy aging in general and background literature on the intersection between migration and health were included to contextualize findings from the narrative review of individual studies.

## Similarities and differences in aging processes across migrants and native populations

Aging encompasses a long time span in the lives of people with associated changes in healthcare needs. In the years prior to retirement, maintaining the ability to work is often a central issue. It is in itself a protective factor against functional decline, morbidity and mortality. In this time span, health behaviour issues relate to maintenance of health and prevention of disease, e.g. participation in screening programmes and preventive health visits. As age increases, issues related to the management of chronic diseases and often multimorbidity, which goes hand in hand with multiple medications, become of higher importance. They comprise timely diagnosis, adherence to treatment and continuity of care, which all necessitate increased interactions with healthcare services and reliance on social support networks that can help to cope with disease. With old age, issues also related to cognitive decline, bereavement and palliative care increase in importance.

Aging processes vary within and across different population groups. They go along with heterogeneity in health status and healthcare needs, which depend on gender, sex, age, socioeconomic background, sociocultural resources, contextual factors at, e.g. community levels, and more distant, structural policies and practices fostering or hindering healthy lives across the lifespan [5, 6, 17, 20]. In the following, we will focus on particular health and healthcare issues faced by aging migrants. To a large extent, they overlap with those faced by socioeconomically disadvantaged aging people in the native populations of the countries they have settled in. Some risks to health occur with age irrespective of migrant background, such as economic disadvantage and decline related to low income after retirement and increased out-of-pocket payments for treatment/medicines; social isolation caused by functional decline, loss of spouse/friends or lack of social roles related to labour market participation; and increased care responsibilities for the spouse [5]. However, other factors shaping aging processes are specific for migrants. This includes exposures in the country of origin, during migration and after settlement [7, 8, 20, 21]. Such factors shape both health behaviours, somatic and mental health and ultimately mortality throughout the life course and thus also at old age [16, 20, 22]. For example, some migrant groups may have been exposed to high rates of infectious diseases, malnutrition and traumatic experiences in the country of origin and/or during migration that influences their life-long risk of illness, further complicated by lack of appropriate preventive care and medical treatment during migration [16, 23]. In addition, migrants are more likely to be exposed to discontinuity of treatment and side effects because of suboptimal adherence due to pendular migration (repeated travels to their country of birth) or during long stays in precarious legal situations (e.g. for asylum seekers and irregular migrants with limited access to healthcare).

Furthermore, migrants from non-Western countries are more likely to be disadvantaged in terms of socioeconomic position due to lower levels of educational achievement, un/underemployment and obligations towards relatives abroad (e.g. through sending of remittances) than native populations [24, 25]. Socioeconomic background is closely related to health behaviours and health status and often has substantial explanatory power in studies of health inequalities between migrants and native populations [19, 26].

In addition, low HDL cholesterol and associated risk of heart disease and intra-abdominal obesity increasing the risk of diabetes exist in some migrant groups. These genetic differences may partly explain higher occurrence of these diseases; however, morbidity differentials are not predetermined but can be reduced by good access to care and uptake of prevention.

Besides migration processes, ethnicity is an additional important factor to take into consideration when describing and explaining health behaviours and health status among aging migrants in Europe [26, 27]. Health behaviours and health status vary substantially across different ethnic groups, often due to a complex interplay between cultural factors, such as dietary practices or physical activity patterns, contextual factors such as socioeconomic background and residential area characteristics (for example, deprivation, inadequate access to services) and individual factors such as language competencies [26, 27].

Language barriers and low health literacy are more common among aging migrants partly due to limited educational experiences and partly to the age-dependent reduction in second language abilities due to cognitive decline. They constitute additional risk factors for poor health and barriers to healthcare that are relatively more common among migrants compared to native European populations. Poor levels of health literacy may lead to less optimal health behaviours, poorer health status and limited access to high quality healthcare [28, 29]. For example, inability to read and act upon written information on how to lower risks for diabetes or how to access a range of medical services may negatively affect the health of aging migrants. Language barriers may as well be even more present among aging refugees suffering from posttraumatic stress syndrome (PTSD) [5, 30].

Psychosocial vulnerability such as experiences with discrimination and segregation in society, stigma pertaining to some diseases and culture-specific perceptions of health and expectations to healthcare constitute additional health-related risk factors and barriers to appropriate, timely and coordinated healthcare for aging migrants [31, 32]. Although most of these factors may place aging migrants in a disadvantaged position in terms of health and healthcare utilization, it is important to also recognize the many sociocultural resources that migrants possess, including the resilience and coping strategies implicit in experiencing migration processes and settling in new contexts and other sociocultural resources such as social support structures in ethnic/religious communities and faith-based or spiritual resources in coping with disease and death [33].

## Specific issues of health and disease among aging migrants

The ability to identify key health needs and efficient strategies for meeting these among aging migrants in Europe is severely restricted by the limited evidence base with few studies reported so far. In appraising the health profiles of aging migrants in Europe, it is furthermore important to acknowledge both the differences within and between groups and importantly that changes do occur with time and across generations as exemplified by studies of changing dietary patterns and sedentary lifestyles among migrant groups settling in Europe [20, 26, 34]. In the following, we briefly outline some specific issues related to health and disease among aging migrants in regard to health and health-seeking behaviours, particular patterns of morbidity and mortality as well as access and quality regarding healthcare.

### Health behaviours and early detection of disease

Health behaviours are largely established prior to old age; however, little is known about this process and potential changes with age among migrants. Aging migrants from non-Western countries may be less likely to drink alcohol whereas aging migrant women may be less likely to smoke compared to native populations [35, 36]. Lack of physical activity, low consumption of fruit and vegetables and intake of a diet high in fat is relatively more common among migrant groups compared to the general native population, however with substantial differences within and between different migrant groups [13, 37]. For screening programmes and other interventions aimed at ensuring early detection of disease among aging adults, there is a substantial social gradient in uptake, of, e.g. breast and colon cancer screening. Some migrant populations are less likely to participate, possibly due to language barriers and differences in risk perceptions. Others, such as Turkish women in Germany, show high participation rates [38, 39]. Of particular relevance for health behaviours among aging migrants are the competing demands they may experience in daily life, making it hard to prioritize health behaviour change and early diagnosis. This relates for example to extended families. While these may comprise a valuable source of practical support in everyday life and for those who are aging and ill, social roles and obligations may as well be a strain on the individual person, in particular in the context of aging [25, 40, 41]. Responsibilities for the well-being of extended families and preferences for upholding care within families may negatively affect the ability to maintain health, for example, through regular physical activity, and to seek healthcare if symptoms arise [25, 40]. Financial obligations, for example, through sending remittances to family members in the country of origin, may be an additional responsibility for aging migrants, thus reducing their financial capability for health-related expenses [25]. Psychosocial vulnerability caused by, e.g. exposure to trauma, social isolation in relation to the wider society or competing priorities within extended family networks, may reduce engagement in health promotion activities and negatively affect the ability to cope with disease, thus shaping health status and healthcare utilization [42]. Even if migrants are part of extended family networks, the availability of supportive networks should not be assumed. Through the process of resettling into Europe, family networks may have been dispersed, sometimes across several countries. Recent migrants, those from smaller ethnic groups and those experiencing functional decline at old age, may find themselves in a situation with few contacts to the wider community [40]. In addition, those with potentially stigmatizing or life-limiting illnesses, such as dementia, cancers or mental illness, may be socially isolated and correspondingly in need of formal social support for example from health and social care professionals and patient associations [41, 43]. Furthermore, research has identified ethnic differences in norms related to family responsibility as some aging migrants prefer to be cared for by their family members rather than professional caregivers [44].

### Particular patterns of morbidity and mortality

Broadly speaking, migrants experience the same morbidity and mortality patterns as the native populations, but with different relative importance, severity and age of onset. Younger migrants may experience a transitory ‘healthy migrant effect’. However, the lower risk of, for example, developing cancers among many migrant groups in Europe is lost with time not only due to changes in health behaviours, such as smoking and sedentary lifestyles, but also due to exposures to different contextual risk factors [20, 45]. It is uncertain whether unfavourable exposures in the country of origin and the immigration country may lead to earlier onset and potentially more severe chronic disease with age among migrants compared to native populations [22, 46]. A recent British study found older people from ethnic minorities to report poorer health outcomes even after controlling for social and economic disadvantages and that health differentials between ethnic minority and the majority population increase with age for both men and women [47]. This supports concerns that whereas native populations may benefit from an increasing compression of morbidity at old age, migrant populations may not benefit in the same way. This would contribute to growing differentials in health between population groups [48]. In terms of disease-specific morbidity, most cancers are less frequent among migrants. Stomach cancer and liver cancer, however, are more common due to low hygiene and infection with *Helicobacter pylori* and hepatitis B/C [49, 50]. For cardiovascular disease, the picture is more heterogeneous and also dependent on the risk in the country of origin. Some populations, in particular of South Asian origin, are at higher risk of both diabetes and cardiovascular disease [27, 49, 51]. As is the case in the general population, and in particularly among aging people, multimorbidity is common among migrant groups in Europe [27, 52]. Some migrant groups have comparably higher rates of gastrointestinal diseases (including esophagitis, gallstone disease, pancreatitis and Crohn’s disease) and higher risks of respiratory disease compared to native populations [53].

Self-rated health is strongly related to future morbidity and mortality and aging migrant and ethnic minority groups have been found to be comparatively more likely to rate their health as poor [47].

The prevalence of poor mental health tends to be higher among migrant groups overall, and specifically depression has been found to be more common among aging migrants across a range of European countries [54–56]. For dementia, no clear pattern emerges, but higher prevalences in some ethnic groups may be related to vascular diseases [56]. Psychological distress is related to a range of individual and contextual factors including stressful situations and events such as experiences of assault, overcrowding, low standards of living and absence of family and confidants that may be more common among migrants of lower socioeconomic background and which may carry particular importance at old age [26, 57]. Suicide tends to be a less common cause of death although suicide rates among aging migrants are increasing [18, 49, 58].

### Access to and quality of healthcare

Access to quality healthcare concerns dimensions related to entitlement, that is, whether these groups have formal rights to services, for example, through comprehensive health insurance schemes, but it also relates to the ability of the individual person to seek appropriate healthcare when in need. Inequities in health and healthcare refer to the presence of systematic and potentially avoidable differences in health status or access to and outcomes of healthcare among population groups defined according to, for example, socioeconomic background, geographical area or ethnicity [59]. Research on access to healthcare among aging migrants in particular is scarce, but health services research focusing on migrants in general has demonstrated that language barriers, low health literacy, lack of social networks facilitating access to timely care and insecurities related to intercultural encounters on part of both patients and providers do constitute barriers that may affect access to care for aging migrants [60, 61]. Inequity in hospital healthcare provision is unlikely to be a major concern in Europe overall [62, 63]. However, access to and quality of rehabilitation programmes was found to be lower among migrants than among the majority population in Germany [64].

## Responding to the increasing number of aging migrants in Europe

Despite the limited evidence base, some overall recommendations for responding to increased ethnic diversity at the policy, practice and research levels can be outlined.

### Migration as a determinant of healthy aging in policy approaches

To ensure equity, societal and healthcare strategies need to be responsive to the aspirations and needs of ethnic diverse populations in an aging Europe. Migration should preferably be acknowledged and incorporated into healthy aging policies such as the European strategy for active and healthy aging as an important determinant of health alongside other key determinants such as gender, age and socioeconomic background. Focus areas for active and healthy aging as outlined by the European Commission include

1. Devising integrated care plans for chronic diseases
2. Ensuring adherence to treatment
3. Delivering personalized health management and falls prevention approaches
4. Preventing frailty and functional decline and ensuring early diagnosis of these condition
5. Scaling-up innovative and evidence-based cases for chronic disease management among aging people
6. Developing independent living solutions, and fostering innovation for age-friendly buildings, cities and environments [1, 3]

Attention to specific needs among aging migrants should be integral in all these strategies to ensure adequate focus and resource allocation.

The needs of aging migrants must become part of organizations’ mainstream activities. Although migration is just one of many factors shaping care and treatment encounters for older people, the process of migration, cultural values and language barriers may give rise to specific needs for aging migrants. There is a need for awareness raising at all institutional levels including cultural sensitivity training of healthcare staff and prioritizing new strategies for overcoming informal barriers in service delivery caused by cultural, socioeconomic and language differences between professionals and aging users [27, 65, 66]. Policy commitment must be combined with relevant and feasible recommendations at the practice level, e.g. pertaining to the need for overcoming language barriers and adapting to cultural differences in healthcare for aging populations. Finally, it is important to ensure accountability and documentation of progress through the use of performance indicators.

### Adapting healthy aging interventions to include aging migrants

Insight into health among diverse migrant groups in Europe has increased substantially in recent decades. However, more focus on particular needs of aging migrants is warranted as we are still lacking robust insight into how to successfully address inequalities in health and living circumstances across aging populations. As health and disease are shaped by a range of exposures and behaviours across the life course of the individual person, there is a need for early interventions depending on the evidence regarding causal processes and the windows of opportunities focusing on promoting and supporting healthy behaviours and cultural resources in migrant groups and preventing risk behaviours that lead to higher disease burden and functional decline among aging people [20, 67]. Although few robust intervention studies have been carried out among aging migrants in Europe to date, general lessons can be teased out based on the knowledge related to principles for public health interventions for ethnic diverse populations [23, 68–70]. In particular, there is a need for combining individual-oriented health interventions focusing on increasing awareness and skills with more comprehensive community-based, structural interventions aiming to ensure that social networks processes, private and public service providers and the built environment of communities facilitate healthy aging that are relevant for ethnic diverse groups of aging people [17, 23, 67]. An inter-sectorial approach is needed when combining interventions at the structural and the individual level, thus necessitating involvement of a range of sectors including primary healthcare, urban planning and non-governmental and private partners such as faith-based associations, religious leaders or private companies in a particular community [6, 17]. Although this is relevant for healthy aging interventions in general, adapting interventions to diverse cultural and language needs is important for successfully addressing the multitude of individual and contextual factors framing the everyday life and aging processes of migrants in an inclusive way [71]. This could be achieved through making interpreters readily available in interventions for diverse groups, and through engaging migrant communities, thereby ensuring relevance of approaches and capacity-building among the target group [71–73]. Intercultural training of front-line staff in contact with aging people, recruitment of ethnic minority staff and volunteers and behaviour change interventions through peer education are examples of such participatory approaches [65, 72]. As aging migrants may be particularly at risk of social isolation, not only strengthening of social support structures within ethnic communities but also establishing relationships to aging people from native populations is important. In addition, mapping relevant public and private stakeholders and gatekeepers in ethnic diverse communities would enable a broader scope in recruitment strategies into healthy aging interventions. Finally, dissemination of good practices across contexts is needed in particular as some European countries have a longer history of adapting healthcare services to culturally and linguistically diverse populations.

A note of caution is warranted as there are differences in needs and resources within and between migrant groups as discussed above. The interactions of many factors in relation to health makes it important to carefully consider whether services for aging migrants should be focused specifically on migrants (migrant-specific services) or just migrant-sensitive (inclusionist) as part of the general healthcare system [74]. The principle of implementing equal strategies in situations of equal needs (horizontal equity) should be balanced with strategies that differ between subpopulations to achieve equal chances of optimum outcomes (vertical equity) in healthy aging. An example of the latter approach is the recently established migrant health clinics at selected Danish hospitals that are specifically targeting the complex healthcare needs among migrant groups and the move towards culturally sensitive care initiatives across a range of European countries [42].

Ensuring timely diagnosis and access to high-quality, well-coordinated patient-centred care is even more central to aging individuals than to the general population, not least within the increasingly complex and accelerated treatment regimens requiring patients to engage in self-care in the context of their everyday life [23, 59, 60]. Besides provision of patient-centred, holistic care within healthcare facilities, culture-sensitive rehabilitation services and psychosocial support for aging patients and their family caregivers must be ensured. This is particularly important in the context of functional and cognitive decline among aging people as the burden on family caregivers, who at times are themselves ill, can be substantial. Migrants may even have different perceptions about caregiving and limited awareness of available sources of practical, emotional and financial support for aging patients [75, 76]. Although the field of palliative or end-of-life care is of importance within aging populations, few studies have explored this topic among migrants. Barriers to the provision of palliative care for migrants have been identified at both the patient and provider levels, pointing to the need for improved awareness and support in case management within healthcare institutions when preparing for increased ethnic diversity in palliative and end-of-life care [41, 77].

### Including migrants in healthy aging research

Our narrative review identified a limited evidence base, with most studies originating from Northern and Western parts of Europe. Migrants need to be more systematically included in empirical research within the field of healthy aging in countries across Europe, thus representing the diversity in policies, service-delivery regimes and contexts framing old age. Aging migrants, especially those with multiple deprivation, are often under-represented in national surveys, and registry studies may be complicated by lacking information on migration status and possibly return bias (the so-called Salmon bias) [12, 78, 79]. There is therefore a need for improved recruitment of ethnic diverse populations with oversampling to ensure representativeness of results and for data collection to be disaggregated also by migration background and socioeconomic status in national registries based on routine data [80]. Comparisons between migrant groups from the same country of origin moving to different host countries, and preferably comparing to disease burdens in their countries of origin, would be helpful in providing insights into the effect of factors specific to immigration countries; however, this requires uniform definition of migrants across Europe as well as attention to differences in disease rates between immigration countries. Longitudinal studies with due consideration of individual, social and contextual factors shaping health at old age are needed. Finally, to facilitate healthy aging interventions that are acceptable and efficient for aging migrants, more intervention studies are needed focusing on both individual- and community-level interventions across the spectrum from health promotion/disease prevention, early detection, adherence to treatment, psychosocial needs among aging people and their relatives and finally end-of-life care for diverse migrant groups. Community-based interventions with inclusion of staff with ethnic diverse backgrounds are recommended. This approach is currently being explored in a Dutch study on the effectiveness of involving ethnic community health workers in the provision of culturally sensitive care for aging migrants [70].

## Conclusions

We have in this paper focused on the health needs and underlying determinants among the growing population of aging migrants in Europe. There is a need for attention to migration and ethnicity alongside other factors such as gender and socioeconomic position that intersect in determining the ability to live a healthy and fulfilling life at old age. The diversity in individual characteristics, life course processes and contextual factors shaping aging processes among migrants necessitates a sensitive and comprehensive approach to policies, practices and research within the field of healthy aging to accommodate for the needs of the growing number of aging migrants in Europe and counter inequities in health and well-being at old age.
